# Outcome of Subsequent Pregnancies in Familial Molar Pregnancy

**Published:** 2013-03-06

**Authors:** Masoumeh Fallahian, Forough Foroughi, Mohammad Vasei, Shahrzad Tavana, Maryam Ghanbary, Maryam Monajemzadeh, Anahita Tavana

**Affiliations:** 1 Department of Obstetrics and Gynecology, Infertility and Reproductive Health Research Center, Shahid Beheshti University of Medical Sciences, Tehran, Iran; 2 Department of Pathology, Shahid Beheshti University of Medical Sciences, Tehran, Iran; 3 Department of Pathology, Tehran University of Medical Sciences, Tehran, Iran; 4 Department of Natural Sciences, University of Texas, Austin, USA

**Keywords:** Hydatidiform Mole, Familial, Outcome of Pregnancy, Normal Pregnancy

## Abstract

Familial recurrent molar pregnancy is an exceedingly rare condition, in which complete hydatidiform moles are mostly diploid but biparental in origin and the outcome of subsequent pregnancies is likely to be a hydatidiform mole or other type of reproductive loss. We previously reported a case of familial molar pregnancy (family K) comprising five affected members (four sisters and one of their cousins) each with at least one hydatidiform mole (HM). In addition to the molar pregnancies, these patients have a total of three miscarriages and 8 normal pregnancies leading to healthy children; but the youngest member of this family has given birth to a boy with Down syndrome.

Our second family (case S) includes two sisters with diploid biparental complete moles. They have a total of six molar pregnancies with no living child. Recently the younger sister had a partial molar pregnancy with apparently normal XX fetus accompanying diffuse molar changes of the placenta that led to preeclampsia and preterm delivery.

Overall, these families have had 26 pregnancies including 12 molar pregnancies (complete or partial) and three abortions.

We concluded that these families are predisposed to various genetic mutations, chromosomal abnormalities and clinical manifestations, which affect their offspring. Further studies of patients are needed to determine any relationship between a history of familial molar pregnancy and trisomy or other chromosomal abnormalities in offspring and genetic mutations in the products of conception to complete the puzzle and manage familial molar pregnancy.

## Introduction

Molar pregnancy is an abnormal pregnancy in which the embryo does not develop or develops abnormally, but proliferation and hydropic degeneration of the placenta villi is seen. Complete hydatidiform moles usually have a 46XX karyotype, and the molar chromosomes are entirely of paternal origin (Androgenic moles). Molar pregnancy in next gestation is rare; Its probability of occurrence is approximately 1% in sporadic partial or complete hydatidiform moles of androgenic origin (1).

Most subsequent pregnancies following a sporadic hydatidiform mole will be full term normal pregnancies. Recurrent molar pregnancy may even be familial, but this is an exceedingly rare condition (2). It is proposed that patients with recurrent hydatidiform moles fall into two groups. Firstly, patients with androgenetic complete moles or triploid partial hydatidiform moles, for whom the risk of a recurrent hydatidiform mole, while raised, is still relatively small after the first hydatidiform mole. Second, patients with biparental complete hydatidiform moles who are most likely to have other complete or partial hydatidiform moles, and are extremely rare (1).

Here we present the outcome of subsequent pregnancies in two cases of familial recurrent hydatidiform.

## Case report

The first case is a member of the family K, whom we previously reported as a case of familial molar pregnancy comprising five affected members, each with at least one hydatidiform mole (3). In addition to the molar pregnancies, these patients had a total of three miscarriages and three normal pregnancies leading to healthy children. Now, three sisters of this family have a total of seven apparently healthy children, while their cousin has not tried to achieve a further pregnancy.

**Fig 1:**
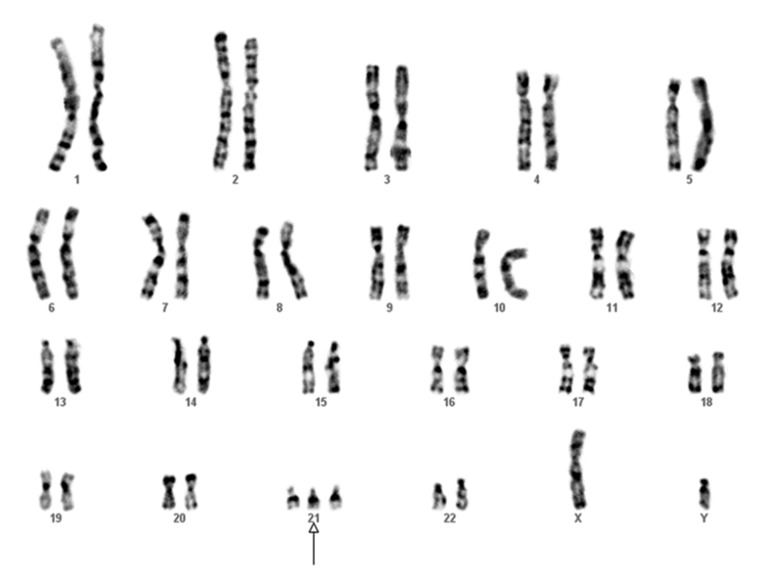
Karyotype of the infant from FRHM family (K) diagnosed with Down syndrome.

Our patient has two children. The first child is a healthy boy, but the second one suffers from congenital heart disease of ventricular septal defect (VSD) and also Down syndrome (Fig 1). The boy had open heart surgery for correction of his disease and is now well. In her past history, the mother had a hydatidiform mole that led to gestational trophoblastic neoplasia. Genetic investigation of this family suggested genetic heterogeneity for familial recurrenthydatidiform moles(FRHM) as linkage and haplotype analysis excluded linkage to 19q13.4, the region where NLRP7, the gene most commonly associated with FRHM, is located. This family provided the first evidence for a second recessive locus responsible for familial molar pregnancies (4).

However, mutation of C6orf221 has not been investigated in this family. The second case is a member of family (S) in which two sisters of the family are affected. The first sister has had recurrent pregnancy loss for four times, including complete molar pregnancies. The younger sister has had two complete molar pregnancies. Genetic analysis of her second molar pregnancy revealed it to be diploid but biparental in origin. Her third pregnancy resulted in a partly molar pregnancy with an apparently normal fetus with XX karyotype in amniocentesis.

This mother had preterm labor that could not be prevented due to signs of preeclampsia (proteinurea 3+ and hypertension (140/90 mmhg)). In addition, the mother had significant manifestations of molar pregnancy, including hyperemesis, hyper thyroidism (higher than normal free T4 and suppressed TSH), theca lutein ovarian cysts, and an enlarged partly molar placenta. At 25-weeks-ofpregnancy an apparently normal female was born but resuscitation was not successful. Amniotic fluid was more than normal (approximately 2 liters), that may be due to large placenta. After delivery, the mother had early post partum hemorrhage and received misoprostol (400 μm, sublingual), but she showed adverse reactions to this medicine with tachycardia (heart rate:120), fever (39˚C), and urticaria. After ruling out thyroid storm, it was controlled by symptomatic treatments.

**Fig 2:**
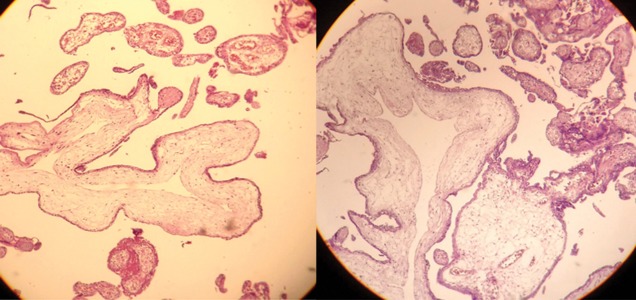
Pathology of partly molar change associated with a normal fetus in family (S).

Samples of placenta and cord blood were preserved for further investigation and the newborn with placenta were sent for pathologic evaluations. Pathology revealed a partial molar pregnancy (Fig 2). Result of cord blood karyotype was 46XX; the placenta also was normal diploid XX. We carried out p57KIP2 immunostaining and the cytotrophoblast cells were clearly positive, consistent with a partial hydatidiform mole.

The mother came back for follow up by monitoring of β-hCG. The level of β-hCG was 64000 mIU/ml at the time of admission and 10 days after delivery it was 550 mIU/ml and now it is negative.

**Fig 3:**
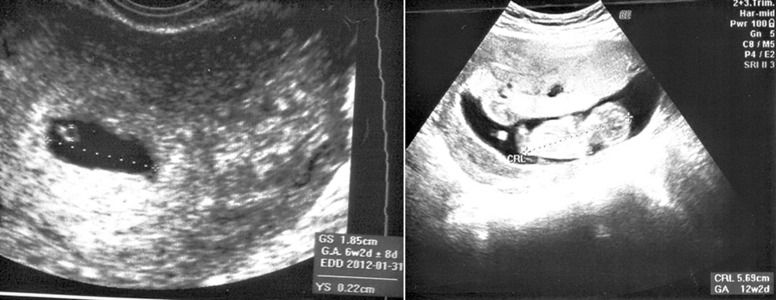
First trimester ultra-sound of partly molar change accompanying a normal fetus in family (S).

Ultrasound on her first trimester, illustrated a singleton pregnancy by an irregular gestational sac. In serial ultra-sound, the placenta has been larger with cystic area sounds like molar changes with large bilateral ovarian cysts, most probably theca lutein cysts (Fig 3). Amniocentesis of amniotic fluid in 17 weeks showed a normal XX female karyotype.

Evaluation of her previous hydatidiform mole (HM). and recent partial moles revealed a diploid complete hydatidiform mole that are biparental in origin suggesting that like her elder sister she has FRHM. Surprisingly both sisters were not found to have a mutation in NLRP7, the gene most common mutated in this condition (5). However, a mutation of C6orf221 was subsequently found in the two sisters (6).

Overall, these families have had 26 pregnancies including twelve molar pregnancies (complete or partial), and three abortions.

## Discussion

Complete hydatidiform mole or androgenic origin usually results from an excess of paternal genomes. In this condition, complete hydatidiform moles are mostly diploid, but biparental in origin and the outcome of subsequent pregnancies is likely to be a hydatidiform mole or other types of reproductive loss (1).

Today, molar pregnancies are assumed to be rare due to early detection of abnormal pregnancies by ultra sound. Nutritional improvement has contributed to the decline of molar pregnancy as well.

Coexistence of molar changes with an apparently healthy fetus is unusual in a case of FRHM. However, it has been reported in other situations, it could be due to a mole and a normal fetus in twin pregnancy, partial mole, partly molar changes, and also mesenchymal dysplasia (7). In our case, as mentioned earlier, the first trimester ultra-sound revealed that it was a singleton pregnancy and the amniocentesis of the fetus ruled out a triploid partial mole. Diffuse molar changes of the placenta affected the outcome of pregnancy. However, our case also demonstrated that both moles were diploid and biparental, not triploid as we expect for a partial mole. This confirmed our genetic examination, in which the fetal tissue was determined to be diploid. There fore, this is a similar situation as seen in other women FRHM, in which the complete moles are diploid and biparental, rather than androgenetic. This fact strongly suggests that the younger sister, like her older sister, has FRHM. While most moles in this condition are complete, affected women do sometimes have other types of reproductive loss, including partial moles.

There are both diverse and heterogeneous causes of FRHM, such as in family K, in which both genetic and environmental factors may interact with causative mutations in FRHM and affect the reproductive outcomes in different pregnancies (even normal full term pregnancy).

With the exception of a mutated C6orf221 in family S, the causes of different pregnancy outcomes in these families have not yet been determined and more investigation is required to clarify these issues. We know that the mutation of NLRP7 is responsible for adverse pregnancy outcomes, however, the mutation was absent in both families. Although environmental and nutritional factors may overcome the effects of other responsible mutant genes, especially in the family K with several term pregnancies, detection of other chromosomal and genetic factors responsible for abnormal offspring should always be taken into consideration. The mutation of C6orf221 has not yet been evaluated in family K.

Since the members of this family mostly have had term pregnancies in their subsequent pregnancies, we can assume that a temporary factor, such as environmental conditions, affect their reproduction in a period of time. In a case-control study, investigators revealed an association between the occurrence of mole hydatidiform pregnancies and the exposure of their husbands to soil and dust (8).

Existence of a chromosomal abnormality (Down syndrome) in the child of one of the members could be coincidental, but we cannot rule out any relationship between genetical and chromosomal abnormalities in FRMH.

Therefore, further studies are required to detect other mutations and integrate the puzzle of causes responsible for familial molar pregnancies in order to manage these adverse reproductive outcomes.

## References

[1] Sebire NJ, Fisher RA, Foskett M, Rees H, Seckl MJ, Newlands ES (2003). Risk of recurrent hydatidiform mole and subsequent
pregnancy outcome following complete or partial
hydatidiform molar pregnancy. BJOG.

[2] Helwani MN, Seoud M, Zahed L, Zaatari G, Khalil A, Slim R (1999). A familial case of recurrent hydatidiform molar pregnancies
with biparental genomic contribution. Hum Genet.

[3] Fallahian M (2003). Familial gestational trophoblastic disease. Placenta.

[4] Slim R, Fallahian M, Rivie`re JB, Zali M R (2005). Evidence of a
genetic heterogeneity of familial hydatidiform moles. Placenta.

[5] Wang CM, Dixon PH, Decordova S, Hodges MD, Sebire NJ, Ozalp S (2009). Identification of 13 novel NLRP7 mutations
in 20 families with recurrent hydatidiform mole; missense
mutations cluster in the leucine rich region. J Med Genet.

[6] Parry DA, Logan CV, Hayward BE, Shires M, Landolsi H, Diggle C (2011). Mutations causing familial biparental hydatidiform
mole implicate c6orf221 as a possible regulator of
genomic imprinting in the human oocyte. Am J Hum Genet.

[7] Sebire NJ, Fisher RA (2005). Partly molar pregnancies that are
not partial moles: additional possibilities and implications. Pediatr Dev Pathol.

[8] Shamshiri-Milani H (2008). Molar pregnancy and husband’s occupation:
do soil and dust have any role?. East Mediterr
Health J.

